# Comparing Efficacy and Safety of Itraconazole Solution Versus Posaconazole for Antifungal Prophylaxis After Heart Transplant

**DOI:** 10.1093/ofid/ofag104

**Published:** 2026-02-28

**Authors:** Ralph Tayyar, Darra Drucker, Roy Lee, Thu Le, Jeff Teuteberg, Kiran Khush, Helen Luikart, Tyler Intrieri, Yasbanoo Moayedi, William Alegria, Thomas Dieringer, Erik Henricksen, Aruna Subramanian

**Affiliations:** Division of Allergy & Infectious Diseases, University of Washington, Seattle, Washington, USA; Division of Pharmacy, Children's Hospital Los Angeles, Los Angeles, California, USA; Department of Transplant, Stanford University, Stanford, California, USA; Department of Transplant, Stanford University, Stanford, California, USA; Section of Heart Failure, Cardiac Transplant, and Mechanical Circulatory Support, Division of Cardiovascular Medicine, Department of Medicine, Stanford University, Stanford, California, USA; Section of Heart Failure, Cardiac Transplant, and Mechanical Circulatory Support, Division of Cardiovascular Medicine, Department of Medicine, Stanford University, Stanford, California, USA; Section of Heart Failure, Cardiac Transplant, and Mechanical Circulatory Support, Division of Cardiovascular Medicine, Department of Medicine, Stanford University, Stanford, California, USA; Section of Heart Failure, Cardiac Transplant, and Mechanical Circulatory Support, Division of Cardiovascular Medicine, Department of Medicine, Stanford University, Stanford, California, USA; Cardiology, University of Toronto, Toronto, Canada; Division of Infectious Diseases & Geographic Medicine, Department of Medicine Stanford University School of Medicine Stanford, Stanford, California, USA; Division of Infectious Diseases & Geographic Medicine, Department of Medicine Stanford University School of Medicine Stanford, Stanford, California, USA; Department of Transplant, Stanford University, Stanford, California, USA; Division of Infectious Diseases & Geographic Medicine, Department of Medicine Stanford University School of Medicine Stanford, Stanford, California, USA

**Keywords:** antifungal prophylaxis, heart transplant, itraconazole, posaconazole

## Abstract

**Background:**

Posaconazole and itraconazole are 2 oral agents that are commonly prescribed for heart transplant recipients requiring *Aspergillus* prophylaxis; however, there are limited data comparing their efficacy and tolerability.

**Methods:**

This was a single-center, retrospective cohort study comparing itraconazole and posaconazole for the prevention of invasive fungal infections in heart transplant recipients between January 2015 and May 2022. The primary efficacy outcome was incidence of fungal infection. The primary safety outcome was incidence of hepatic dysfunction after initiation of therapy. Standard duration of prophylaxis therapy was 3 months for both cohorts.

**Results:**

A total of 134 patients received itraconazole prophylaxis and 98 received posaconazole prophylaxis. Patients receiving itraconazole were more likely to have received induction immunosuppression (*P* < .001) and more likely to have received a heart-kidney transplant, but other baseline characteristics were similar. Patients receiving itraconazole prophylaxis had a higher rate of fungal infection (7.4% vs 0%, *P* & .0065) and were more likely to have therapy changed to an alternative antifungal (20.9% vs 10.2%, *P* & .03). Adverse events were similar between cohorts.

**Conclusions:**

Posaconazole prophylaxis after heart transplant was associated with a lower rate of fungal infections and reduced need to change to alternative therapy when compared to itraconazole.

## Teaser

Posaconazole prophylaxis in heart transplant recipients resulted in fewer invasive fungal infections and fewer therapy changes compared with itraconazole, with similar adverse events.

Heart transplant (HT) recipients are at increased risk of infection due to receiving immunosuppressive therapy to reduce the risk of allograft rejection [1, 2]. Aspergillosis is the most common invasive mold infection in solid-organ transplant recipients, with incidence ranging from 1.7% to 14% (average 6%) and the mortality attributed to aspergillosis is up to 67% in HT recipients. The American Society of Transplantation recommends targeted *Aspergillus* prophylaxis with itraconazole, voriconazole, or echinocandin in patients with additional risk factors, such as thoracic reoperation, cytomegalovirus disease, and posttransplant hemodialysis [1, 3].

Posaconazole has been shown to be noninferior to voriconazole for the treatment of invasive aspergillosis and has been used by some transplant centers as prophylaxis in patients after solid-organ transplant [4].

Itraconazole and posaconazole are both associated with hepatotoxicity [5, 6], but in studies comparing itraconazole and posaconazole as antifungal prophylaxis following allogeneic hematopoietic stem cell transplant, there was no significant difference in the percentage of potential drug-related adverse events between the 2 broad-spectrum triazole agents [7, 8]. Beyond Mucorales, posaconazole shows broader and more potent activity than itraconazole against *Candida* spp. (generally 2–4 fold lower minimum inhibitory concentration, including improved activity against *Candida glabrata*) and *Cryptococcus neoformans* [9]. Moreover, posaconazole has comparable or slightly superior activity against *Aspergillus* spp., with retained activity against some itraconazole-resistant *A fumigatus* isolates and modestly better activity against select non-*Aspergillus* molds such as *Scedosporium* and some *Fusarium* spp [10, 11]. However, a recent study comparing these 2 antifungal agents as systemic prophylaxis in critically ill lung transplant recipients found fewer fungal infections in patients on posaconazole compared to those on itraconazole [12].

Although previous studies have investigated antifungal prophylaxis in hematopoietic stem cell and lung transplant recipients, there are limited comparative data on the safety and efficacy of itraconazole and posaconazole as antifungal prophylaxis after HT. Historically, our center experienced an increase in non-*Candida* fungal infections, which resulted in addition of systemic antifungal therapy being given to our heart transplant recipients. Given the change in our center's postoperative HT *Aspergillus* prophylaxis protocol from itraconazole to posaconazole, we aimed to compare the relative safety and efficacy of these 2 agents after heart transplantation. The protocol change was implemented to improve patient quality of life (due to difficult administration of itraconazole solution), expanded availability of oral anticoagulant options in patients with kidney dysfunction when treated with posaconazole, and the reduced cost of generic formulations of posaconazole [13].

## MATERIALS AND METHODS

This was a single-center, retrospective cohort study at Stanford Health Care comparing itraconazole and posaconazole for the prevention of invasive fungal infections in HT recipients from January 2015 through May 2022. Patients were followed for 12 months posttransplant. Data were manually collected through chart review from the electronic medical record. The antifungal per protocol was itraconazole from 2015 through 2019, and posaconazole from 2020 forward. However, patients were not excluded if they received itraconazole or posaconazole outside of their protocol cohort. The Stanford Institutional Review Board approved the study. Patients at least 18 years of age were eligible if they were initiated on itraconazole or posaconazole posttransplant for *Aspergillus* prophylaxis. Patients were excluded if they received a combined heart-lung or heart-liver transplant, transitioned to another institution within the first year posttransplant, expired within 7 days of heart transplant, or were initiated on an alternative antifungal agent posttransplant.

At our institution, patients receive three months of antifungal prophylaxis with a triazole antifungal agent, as well as inhaled amphotericin b as adjunctive mold prophylaxis during their index hospitalization. Patients initiated on posaconazole received 300 mg daily, and those initiated on itraconazole received 200 mg daily in the morning and 100 mg in the evening, along with nystatin oral suspension. Use of posaconazole oral solution and itraconazole oral tablet formulations was avoided because of poor and variable bioavailability. Therapeutic drug levels for prophylaxis were defined as greater than 0.7 mcg/mL for posaconazole and greater than 0.5 mcg/mL for itraconazole [14, 15].

The primary efficacy outcome was the incidence of fungal infections posttransplant, defined by the 2020 consensus definitions of invasive fungal disease by the European Organization for Research and Treatment of Cancer and the Mycoses Study Group Education and Research Consortium [16]. Proven, probable, and possible invasive fungal infections were included. Infection-free survival through the first year after initiation of antifungal prophylaxis was evaluated as an additional efficacy outcome. The primary safety outcome was incidence of hepatic injury, defined as elevations in liver transaminases greater than 200 IU/mL (about 5 times the upper limit of normal) [17]. Secondary outcomes included elevations in liver transaminases above 150 IU/mL (about 3 times the upper limit of normal), incidence of prolonged corrected QT interval (QTc) greater than 500 ms within 2 weeks and 3 months after initiation of the antifungal agent, percentage of switch in antifungal prophylaxis agents, percentage of discontinuation of antifungal prophylaxis earlier than the planned 3-month course, median duration of antifungal prophylaxis, and the median time to therapeutic antifungal serum drug levels. Laboratory tests including aspartate aminotransferase (AST), alanine aminotransferase (ALT), alkaline phosphatase, and total bilirubin were evaluated at baseline (before antifungal agent initiation), 14 ± 3 days after antifungal agent initiation, and at the maximum value within the first 14 ± 3 days after antifungal agent initiation. Concomitant medications commonly associated with drug-induced liver injury administered within 2 weeks of antifungal agent initiation were recorded and included: amoxicillin-clavulanate, disulfiram, diclofenac, carbamazepine, ibuprofen, erythromycin, HMG CoA reductase inhibitors, and anabolic steroids. Additionally, concomitant medications commonly known to prolong the QTc interval administered within 2 weeks of antifungal agent initiation were recorded and included: ondansetron, fluoroquinolone and macrolide antibiotics, amiodarone, methadone, sumatriptan, metoclopramide, sotalol, dofetilide, antipsychotics, and antidepressants [18, 19].

Chi-squared and Fisher exact tests were used to analyze categorical data, and the *t*-test was used to evaluate continuous data. The level of significance (alpha) was set at 0.05. Descriptive data were reported as medians with interquartile ranges. We used the Kaplan-Meier method to evaluate infection-free survival within the first year after initiation of antifungal prophylaxis and time to discontinuation of antifungal prophylaxis. Data were collected using the online REDCap data management tool [20, 21]. Statistical analysis was performed using R studio version 4.3.3.

## RESULTS

A total of 472 patients were screened for inclusion into this study. After excluding 14 patients who received a heart-liver transplant, 213 patients who transitioned care to another institution, and 13 patients who received an alternative or no antifungal prophylaxis agent, 232 patients were included in the analysis, of whom 134 received itraconazole and 98 patients received posaconazole. Table 1 shows baseline patient characteristics. Patients receiving itraconazole were significantly more likely to have been induced with antithymocyte globulin (ATG) than patients receiving posaconazole (88.8% vs 58.2%, *P* < .001), and there was a significantly higher percentage of heart-kidney transplants in patients receiving itraconazole compared to those receiving posaconazole (13.4% vs 5.1%, *P* = .045). Otherwise, baseline characteristics were similar. The median age was 55 years (interquartile range, 46–61) in patients receiving itraconazole and 46 years (interquartile range, 47–62) in patients receiving posaconazole, and majority of patients were male, 71.6% and 77.6% in patients receiving itraconazole and posaconazole, respectively. The most frequent underlying disease leading to heart transplantation was dilated cardiomyopathy.

**Table 1.** Baseline Characteristics

Baseline Characteristic	Itraconazole (n = 134)	Posaconazole (n = 98)	*P* Value
Male sex, no. (%)	96 (71.6)	76 (77.6)	.36
Race, no. (%)			.11
White	73 (54.5)	54 (55.1)	
Black or African American	11 (8.2)	12 (12.2)	
Asian	20 (14.9)	14 (14.3)	
Native Hawaiian or Other Pacific Islander	8 (6.0)	0 (0)	
Unknown/Other	22 (16.4)	18 (18.4)	
Ethnicity, no. (%)			1
Hispanic/Latino	25 (18.7)	18 (18.4)	
Non-Hispanic or Latino	109 (81.3)	80 (81.6)	
BMI (kg/m^2^), median (IQR)	27.2 (24.3–31.0)	27.3 (23.9–30.8)	.95
Antithymocyte globulin use, no. (%)	119 (88.8)	57 (58.2)	<.001
Transplant type, no (%)			.045
Heart	116(86.6)	93 (94.9)	
Heart-kidney	18 (13.4)	5 (5.1)	
Primary transplant diagnosis			.75
Congenital heart disease	6 (4.5)	5 (5.1)	
Dilated cardiomyopathy	71 (53.0)	46 (46.9)	
Ischemic cardiomyopathy	31 (23.1)	23 (23.5)	
Other	26 (19.4)	24 (24.5)	
Initial drug level therapeutic, no. (%)	21 (15.7)	80 (81.6)	<.001

Abbreviations: BMI, body mass index; IQR, interquartile range.

**Figure 1.**
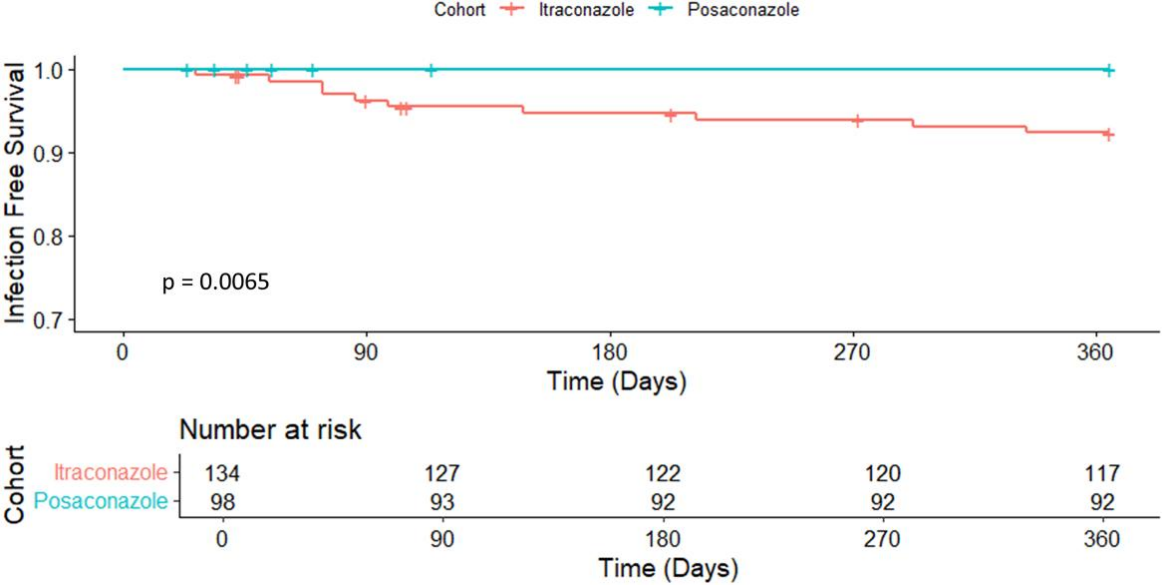
Fungal infection-free survival within 1 year after transplant.

A total of 8 (6%) breakthrough fungal infections were observed in the itraconazole cohort compared to 0 (0%) infections in the posaconazole cohort (Supplementary Table 1). Patients receiving itraconazole were significantly more likely to develop a fungal infection within a year after transplant (*P* & .014) (Figure 1). Of the 8 observed breakthrough infections, 7 patients received induction with antithymocyte globulin (88%). Two additional late fungal infections occurred 292 and 334 days after transplant, both in the itraconazole group. In a Firth penalized logistic regression adjusting for triazole therapy, race, induction therapy, and transplant type, posaconazole prophylaxis was independently associated with significantly lower odds of 1-year fungal infection (odds ratio, 0.08; 95% confidence interval, ∼0.001–0.72; *P* = .019). No other covariates demonstrated statistically significant associations.

There was no significant difference observed in elevations in ALT > 200 IU/mL (*P* & 1.0) or AST > 200 IU/mL (*P* & .135) 2 weeks after antifungal initiation between patients receiving itraconazole and posaconazole (Table 2). Similarly, incidence of elevations in ALT > 150 IU/mL (*P* = 1.0) or in AST > 150 IU/mL (*P* = .4703) was similar between the cohorts 2 weeks after antifungal initiation. Itraconazole was associated with significantly higher rates of switching antifungal prophylaxis agents in comparison to posaconazole (*P* = .03), most commonly because of subtherapeutic antifungal levels (9%), concern for infection (6%), and adverse effects (2.9%). There was no observed statistical difference in the early discontinuation of antifungal prophylaxis before 3 months from adverse effects (*P* > .05).

**Table 2.** Characteristics of Posaconazole and Itraconazole Therapies

Outcomes	Itraconazole (n = 134)	Posaconazole (n = 98)	*P* Value
Primary safety outcome			
AST > 200 IU/mL, no. (%)	14 (10.4)	6 (6.1)	.25
ALT > 200 IU/mL, no. (%)	16 (11.9)	8 (8.2)	.35
Secondary safety outcome			
AST > 150 IU/mL, no. (%)	22 (16.4)	10 (10.2)	.18
ALT > 150 IU/mL, no. (%)	21 (15.7)	11 (11.2)	.33
Early discontinuation of antifungal prophylaxis, no. (%)			.68
Total	16 (11.9)	10 (9.6)	
Elevated LFTs	5 (3.7)	3 (3.1)	
Cost	1 (0.7)	1 (1.0)	
Clinical condition	3 (2.2)	2 (2.0)	
Formulation	0 (0)	1 (1.0)	
Drug interaction	2 (1.5)	1 (1.0)	
Alternative infection	1 (0.7)	0 (0)	
Unknown/other	4 (2.9)	1 (1.0)	
Converted to alternative antifungal agent, no. (%)			
Total	28 (20.9)	10 (10.2)	.03
Adverse effect	4 (2.9)	0 (0)	
Subtherapeutic antifungal levels	12 (9.0)	0 (0)	
Concern for Infection	8 (6.0)	0 (0)	
Insurance cost/access/adherence	3 (2.2)	3 (3.1)	
Drug shortage	0 (0)	2 (2.0)	
Formulation	1 (0.7)	4 (4.1)	
Drug interaction	1 (0.7)	0 (0)	
Unknown/other	1 (0.7)	1 (1.0)	
QTc prolongation within 2 wks after antifungal initiation, no. (%)	16 (11.9)	6 (6.1)	.14

Abbreviations: ALT, aspartate aminotransferase; AST, alanine aminotransferase; LFT, liver function tests; QTc, corrected QT interval.

A higher percentage of posaconazole drug levels were therapeutic at initial check compared to itraconazole drug levels (81.6% vs 52.6%, *P* < .001). Twenty patients receiving itraconazole (14.9%) and 14 patients receiving posaconazole (14.3%) had no therapeutic antifungal drug levels obtained.

## DISCUSSION

To the best of our knowledge, this single-center, retrospective cohort study comparing the efficacy and safety of itraconazole and posaconazole for *Aspergillus* prophylaxis is the first investigation of these agents used after heart transplantation. Our study demonstrated posaconazole to be significantly more effective in the prevention of fungal infection after HT in comparison to itraconazole. These results are similar to previous findings from a study comparing posaconazole and itraconazole for antifungal prophylaxis in lung transplant recipients [9]. Additionally, significantly more patients receiving itraconazole switched to an alternative antifungal agent than those receiving posaconazole because of concern for infection.

Significantly more patients receiving itraconazole prophylaxis received ATG induction compared to patients receiving posaconazole prophylaxis. This could be a confounding factor because ATG can increase patient's risk of infection. One randomized trial analyzing data from the United Kingdom Cardiothoracic Transplant Audit including 1143 heart transplant recipients induced with ATG reported 18% higher number of infection episodes of any type, in the first year after transplant in the ATG group compared to those who did not receive ATG, and this difference was statistically significant [22].

Among patients receiving itraconazole who developed infectious complications, only 2 breakthrough infections were caused by *Aspergillus*. Itraconazole and posaconazole are both active against *Aspergillus*, though posaconazole provides additional benefit with added activity against non-*Aspergillus* species, including mucoromycetes [5, 6].

Similar to findings in previous studies comparing itraconazole and posaconazole in hematopoietic stem cell transplantation [7], our results demonstrate no significant difference in safety outcomes of these 2 antifungal agents, including drug-drug interactions with transplant medications [23].

The various formulations of posaconazole and itraconazole demonstrate differences in drug absorption, and our center preferentially uses posaconazole oral tablets and itraconazole oral solution. Original oral itraconazole formulations are 55% bioavailable, and itraconazole oral solution is associated with greater exposure in comparison to itraconazole oral capsules. However, high interpatient variability in itraconazole exposure is observed for these formulations and can be affected by concomitant food administration and stomach acidity [5]. Oral formulations of posaconazole include an oral solution and delayed-release tablets. Posaconazole delayed-release tablets are more bioavailable and are less affected by stomach acidity and gut motility in comparison to the solution formulation. Similar to the itraconazole formulations, the posaconazole formulations demonstrate high interpatient variability [6, 24]. Our study showed a higher percentage of posaconazole drug levels were therapeutic compared to itraconazole drug levels and a significantly shorter time to therapeutic drug levels was achieved with posaconazole compared to itraconazole. These differences in therapeutic antifungal levels may also contribute to the higher incidence of fungal infections in patients receiving itraconazole. In our study, liquid formulation was used for all patients, and counseling to take on an empty stomach was provided around the time of transplant. We believe part of the associated lower rates of fungal infections in the posaconazole cohort is likely due to patients being therapeutic for a greater amount of time with improved pharmacokinetics of posaconazole.

Recently a new formulation of itraconazole has become available in the United States with improved bioavailability, and may result in improved efficacy, but as a generic formulation is not available, this therapy may be cost prohibitive for many patients.

Our study has several limitations. External validity of results is limited by the single-center design of this study and relatively small sample size. Additionally, bias related to unobserved confounders cannot be excluded due to the retrospective study design. Last, although this study attempted to account for confounding concurrent medications that can cause similar adverse effects to those caused by itraconazole and posaconazole, including medications that can cause hepatic injury and prolong QTc intervals, the lists of these medications were not exhaustive.

## CONCLUSION

The use of posaconazole as antifungal prophylaxis for heart transplant recipients within the first three months of transplantation was associated with significantly improved fungal infection-free survival at 1 year posttransplant and fewer fungal breakthrough infections compared to itraconazole solution. Itraconazole and posaconazole demonstrated comparable safety for this indication.

## Supplementary Material

ofag104_Supplementary_Data

## Supplementary Data

Supplementary materials are available at *Open Forum Infectious Diseases* online. Consisting of data provided by the authors to benefit the reader, the posted materials are not copyedited and are the sole responsibility of the authors, so questions or comments should be addressed to the corresponding author.
