# Supplementary material for: Comparing Efficacy and Safety of Itraconazole Solution Versus Posaconazole for Antifungal Prophylaxis After Heart Transplant
Source: Open Forum Infect Dis. 2026 Feb 28;13(4):ofag104. doi: 10.1093/ofid/ofag104 (PMC13034668; doi:10.1093/ofid/ofag104)
Supplement: ofag104_Supplementary_Data [file ofag104_supplementary_data.zip › Supplementary table 1.rtf]

Supplementary Table 1. Observed Breakthrough Fungal Infections
Therapy	Organism	Location	Time to Infection (days)	Drug Level (mcg/mL	Drug Level Therapeutic?	
Itraconazole	Cryptococcus	Skin/soft tissue	212	0.1 	No	
Itraconazole	Candida orthopsilosis	Boin/joint	54	0.2	No	
Itraconazole	Aspergillus fumigatus	Lung	148	Undetectable x2	No	
Itraconazole	Aspergillus fumigatus, Aspergillus terreus	Lung	74	0.1	No	
Itraconazole	Culture negative, nodules on imaging improved after starting posaconazole	Lung	98	0.1 initial, 0.3 at time of presumed fungal pneumonia	Initial no, at time of suspected infection yes	
Itraconazole	Culture negative, presumed fungal infection by imaging with mortality	Lung	74	Undetectable x2, 0.3 most recent prior to presumed infection	No x2, at time of suspected infection yes	
Itraconazole	Candida albicans	Bone/joint	27	1.0	Yes	
Itraconazole	Candida albicans	Blood	86	0.3	Yes	
